# Effect of chain architecture on the compression behavior of nanoscale polyethylene particles

**DOI:** 10.1186/1556-276X-8-322

**Published:** 2013-07-15

**Authors:** Jianyang Wu, Jianying He, Gregory M Odegard, Zhiliang Zhang

**Affiliations:** 1 NTNU Nanomechanical Lab, Norwegian University of Science and Technology (NTNU), Richard Birkelands vei 1a, Trondheim 7491, Norway; 2 Department of Mechanical Engineering - Engineering Mechanics, Michigan Technological University, 1400 Townsend Drive, Houghton, MI 49931, USA

**Keywords:** Chain architecture, Nanoscale particle, Spherical hydrostatic compression, Compression properties, Flat-punch MD simulation

## Abstract

Polymeric particles with controlled internal molecular architectures play an important role as constituents in many composite materials for a number of emerging applications. In this study, classical molecular dynamics techniques are employed to predict the effect of chain architecture on the compression behavior of nanoscale polyethylene particles subjected to simulated flat-punch testing. Cross-linked, branched, and linear polyethylene chain architectures are each studied in the simulations. Results indicate that chain architecture has a significant influence on the mechanical properties of polyethylene nanoparticles, with the network configuration exhibiting higher compressive strengths than the branched and linear architectures. These findings are verified with simulations of bulk polyethylene. The compressive stress versus strain profiles of particles show four distinct regimes, differing with that of experimental micron-sized particles. The results of this study indicate that the mechanical response of polyethylene nanoparticles can be custom-tailored for specific applications by changing the molecular architecture.

## Background

Polymers play an indispensable and ubiquitous role in daily life. One approach to produce high-performance or multifunctional polymer materials is to blend chemically different monomers, add advanced fillers, and synthesize specific molecular architectures. It is well known that varying molecular architecture through branching and networking strongly influences the mechanical, dielectric, and thermal properties of polymers. For example, cross-linked molecular architectures enhance the strength and modulus of polymers but generally reduce their fracture toughness [1-3]. However, it has been recently shown that polymer hydrogels that form ionically and covalently cross-linked networks and have fracture energies of 9,000 J/m^2^ can withstand stretches of over 20 [4]. Thus, tuning the molecular architecture can provide opportunities to custom-tailor polymer material properties for specific applications.

On the other hand, polymers at nanoscale dimension are a novel class of materials that offer diverse properties, which can be distinguished from their bulk counterparts. Nanoscale polymeric particles have attracted extensive attention from both the scientific community and industry. Polymeric nanoparticles are featured prominently in a wide variety of applications such as toners, coatings, adhesives, instrument calibration standards, column packing materials for chromatography, biomedicine, and biochemical analysis [5-7]. An emerging application focuses on metal-coated conductive polymeric particles for anisotropic conductive adhesives used in liquid crystal displays and microsystems. The use of these particles could reduce package sizes and manufacturing costs and entirely eliminate the use of lead in these systems [8-11]. The continued expansion of polymeric nanoparticles to new applications has revealed unexpected behaviors and potential shortcomings. Therefore, a complete understanding of their properties is of great importance for their successful use.

Most of the previous research on nanoscale polymers have been focused on properties of thin polymer films due to their relatively easy preparation, characterization, and established applications. It has been explicitly shown that the glass transition temperature (*T*_g_) of polymer thin films is reduced from that of the bulk due to the presence of a free interface, and *T*_g_ is found to be strongly dependent on the film thickness and chain architecture [12-15]. Several studies have been conducted on the thermal properties of polymeric particles and reached similar conclusions as with thin films [16-18]. However, few studies have been performed on the mechanical characterization of freestanding polymeric nanoparticles because of their small size and spherical geometry. Recently, a nanoindentation-based flat-punch experimental technique was developed to characterize the mechanical properties of isolated micron-sized polymeric particles [19,20]. The mechanical response was shown to be highly dependent on the particle size and cross-link density [21,22].

A limited number of computational studies have been carried out to investigate structure and properties of polymeric nanoparticles at the molecular level. Fukui et al. [23] developed a method based on molecular dynamics (MD) to generate polymeric nanoparticle models with linear chain architectures in a layer-by-layer manner. Their results indicated that structural and thermal properties are dependent on particle size. Hathorn et al. [24] investigated the dynamic collision of polyethylene (PE) nanoparticles containing linear molecular architectures. Very recently, our group has studied the effect of size on the mechanical properties of PE nanoparticles via coarse-grained MD simulation (Zhao JH, Nagao S, Odegard GM, Zhang ZL, Kristiansen H, He JY: Size-dependent mechanical behavior of nanoscale polymer particles through coarse-grained molecular dynamics simulation. submitted). Although these pioneering experimental and simulation works have provided insight into the macroscopic properties of polymeric particles, fundamental knowledge of molecular-level behavior is still missing, particularly for different chain architectures.

In this paper, a novel method to construct MD simulation models of ultrafine and stable PE nanoparticles with different molecular architecture is introduced. The MD models are used to examine the compressive flat-punch behavior of PE nanoparticles with linear, branched, and cross-linked chains. It is shown that the chain architecture has a significant effect on the compression behavior of freestanding individual PE nanoparticles.

## Methods

A combination of united-atom force fields [25-28] was used for the MD models of polymeric nanoparticles in which the CH, CH_2_, and CH_3_ groups were considered to be single spherical neutral interacting beads, resulting in great saving in terms of the total number of atoms in the simulated systems. Each of these united-atom models has been shown to be applicable to entangled linear and branched PE polymer systems. The total potential energy can be expressed as:

(1) $$E_{\text{total}}=E_{\mathrm{nb}}+E_{\text{bond}}=E_{\mathrm{nb}}+E_{b}+E_{\theta }+E_{\varphi },$$

where the total potential energy (*E*_total_) includes two components: non-bonded (*E*_nb_) and bonded (*E*_bond_) interaction terms. For the non-bonded interaction term, all the inter-beads separated by more than three bonds only interact through a standard 12–6 Lennard-Jones potential. The cutoff distance was set to 12 Å in the simulations. Standard Lorentz-Berthelot's combining rules were utilized for the unlike-pair interactions. The bonded term comprises three contributions: bond stretching (*E*_b_), angle bending (*E*_θ_), and dihedral torsion (*E*_φ_), in which dihedral torsion is expressed by a cosine polynomial and bond stretching and angle bending are described by harmonic functions. The detailed potential function forms and their respective parameters are summarized in Table 1.

**Table 1:** Potential functions and parameters of united atom force field

**Non-bond**				**Bond**			**Angle**			**Torsion**				
$$E_{\mathrm{nb}}=\left(\begin{array}{cc} 4\epsilon \left[{\left(\frac{\sigma }{r}\right)}^{12}-{\left(\frac{\sigma }{r}\right)}^{6}\right] & ,r\;<\;r_{c}\\ 0 & ,r\;\geq \;r_{c} \end{array}\right)$$				$E_{b}=\frac{1}{2}k_{b}{\left(r_{b}-r_{0}\right)}^{2}$			$E_{\theta }=\frac{1}{2}k_{\theta }{\left(\theta -\theta_{0}\right)}^{2}$			$E_{\phi }=\sum_{n=0}^{3}A_{n}\cos^{n-1}\left(\phi \right)$				
														
	***ϵ *(kcal/mol)**	***σ *(Å)**	***r*c (Å)**		***k***_**b **_**(kcal/(mol·Å**^**2**^**))**	***r***_**0 **_**(Å)**		***k***_**θ **_**(kcal/mol)**	***θ***_**0 **_**(deg)**		***A***_**0 **_**(kcal/mol)**	***A***_**1 **_**(kcal/mol)**	***A***_**2 **_**(kcal/mol)**	***A***_**3 **_**(kcal/mol)**
CH_*x*_… CH_*y*_ (*x* = 1, 2, 3; *y* = 2, 3) [25]	0.1119	4.01	12	CH_*x*_-CH_*y*_	95.89	1.54	CH_*x*_-CH_2_-CH_*y*_	57.6	111.6	CH_*x*_-CH_2_-CH_2_-CH_*y*_	1.73	−4.493	0.776	6.99
				(*x*, *y* = 1, 2, 3) [27]										
							(*x*, *y* = 1, 2, 3) [27]							
										(*x*, *y* = 1, 2, 3) [25]				
CH… CH [26]	0.0789	3.85	12				CH_*x*_-CH-CH_*y*_	62.1	109.74	CH_*x*_-CH-CH_2_-CH_*y*_	0.8143	1.7926	0.3891	3.6743
							(*x*, *y* = 2) [26]							
										(*x*, *y* = 2) [28]				

**Figure 1:**
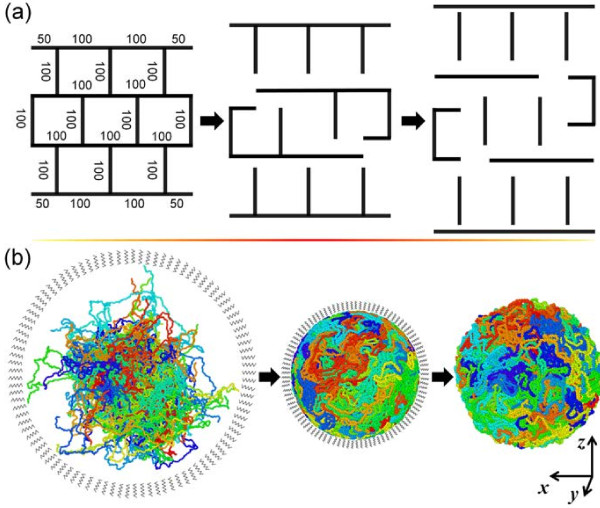
**Unit molecular network structure and schematic depiction of PE particles. (a)** Unit molecular network structure of polyethylene (PE). A networked molecule C_2200_ is decomposed into branched and linear molecules via bond breaking at cross-linking points. The number of united atoms in each linear segment is indicated. The beads at the ends of as-generated branched and linear molecules are hence re-defined (from CH to CH3 bead). **(b)** Schematic depiction of the preparation of ultrafine nanoscale PE particles. PE molecules are packed into a spherical shape via shrinking under hydrostatic pressure. The as-generated nanoparticle is able to maintain the spherical shape under full relaxation. Each simulated bulk or particle system consists of 66,000 beads in total. Coloring of beads is based on the molecule number.

Three distinct PE molecule structures were constructed to study the effect of chain architecture on the mechanical behavior. Figure 1a shows a schematic of the cross-linked, branched, and linear chains that were constructed using the united atoms. For each of the three PE systems, an MD simulation box with periodical boundary conditions was built based on the method of Theodorou and Suter [29]. Each simulation box had an initial bulk density of 0.5 g/cm^3^ composed of 30 of the corresponding systems shown in Figure 1a. MD simulations were run for 200 ps under constant mole, volume, and temperature (NVT) ensemble at 1,000 K controlled by Nosé-Hoover thermostat [30] to allow the molecular structures to relax. Following this, 200-ps constant mole, pressure, and temperature (NPT) runs were conducted at the same temperature and zero pressure in three directions using the Nosé-Hoover thermostat and barostat [30,31]. The bulk systems were subsequently cooled down to 50 K at a rate of 4.75 K/ps with zero external pressure under NPT ensemble. After a short NPT run for 50 ps at 50 K, the systems are heated to 600 K with a rate of 1.1 K/ps, and the density of the bulk systems were monitored during the heating process. The systems were subsequently cooled down from 600 to 200 K at a rate of 2 K/ps. Finally, two steps of relaxation were performed under NPT and NVT ensembles with 100 ps each to obtain samples for mechanical load simulations. These MD models are henceforth referred to as the bulk MD models.

MD models of PE nanoparticles were constructed as shown in Figure 1b. The periodic boundary conditions of the bulk MD models were removed in all directions, and a spherical wall was introduced to encircle all the beads. The beads falling outside the circle will be dragged into the circle. The spherical wall was able to exert a force onto each atom with the magnitude defined by:

(2) $$F\left(r\right)=\left\{\begin{array}{l} -K{\left(r-R\right)}^{2},\;r\;\geq \;R\\ 0\;,\;r\;<\;R \end{array}\right)$$

where *K* is a specified force constant which is given to 5.0 kcal/(moleÅ^2^), *r* is the distance from the bead to the center of the sphere, and *R* is the radius of the sphere. The negative magnitude of the force in Equation 2 indicates that the force acts towards the center of the sphere. Therefore, higher pulling forces are applied to beads far away from the edge of the sphere. The radius of the sphere was reduced to densify the polymer as described by:

(3) $$R=R_{0}S^{n},$$

where *R* and *R*_0_ are the instantaneous and initial radius of the spherical wall, respectively, *S* is a positive constant, and *n* has progressive values of positive integers corresponding to elapsed time of the simulation (i.e., *n* = 1, 2, 3, …). For the simulations described herein, *S* was 0.99 and *n* increased by a value of 1 for every 5 ps of simulation time. Prior to this dynamic relaxation, the confined PE models were first quasi-statically relaxed to a local minimum potential energy configuration using the conjugate gradient method. For each increment of the subsequent dynamic compression, the systems were simulated in the NVT ensemble at 1,000 K, and the density of the polymeric particle was monitored. When the density reached 1.0 g/cm^3^, the compression was terminated. The confined nanoparticle were annealed at 1,000 K for 200 ps to reach a favorable energy configuration and then cooled down to 50 K at a rate of 2.375 K/ps in the absence of the spherical wall. The isolated nanoparticle was heated to 600 K at a rate of 1.1 K/ps, followed by cooling down to 200 K at a rate of 2 K/ps. Finally, 200 ps NVT runs were performed for relaxing the system, and the ultrafine PE nanoparticles were complete.

## Results and discussion

**Figure 2:**
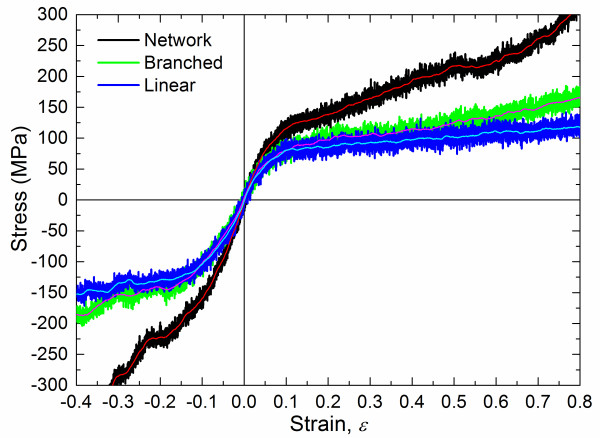
**Tensile and compressive stress versus strain curves of bulk PE with three distinct chain architectures.** Thin lines denote the mean of the bold.

Uniaxial tension/compression simulations were performed on the bulk PE MD models under deformation control conditions with a strain rate of 0.000133/ps at *T* = 200 K in the NPT ensemble based on the Nosé-Hoover thermostat and barostat [30,31]. The lateral faces were maintained at zero pressure to simulate the Poisson contraction. The Nosé-Hoover style non-Hamiltonian NPT equations of motion were described in detail by Shinoda et al. [32]. Figure 2 shows the resultant tensile and compressive stress–strain responses of the three different chain architectures. Initially, each of the responses is stiff and linear but evolves to nonlinear behavior close to a strain of 0.025. Both tension and compression stresses continue to increase in magnitude in a nonlinear manner for the entire range of the simulated deformations. Young's moduli were calculated from a linear fit to the curves within strain of 0.025 and are listed in Table 2. These values indicate that the network modulus is significantly higher than the linear or branched moduli. Similarly, the yield strength appears to be significantly higher for the network material relative to the linear and branched systems. Therefore, it is clear that cross-linking significantly enhances the mechanical properties of amorphous PE.

**Table 2:** Tensile and compressive modulus of bulk and particle PE with different chain architectures

**Chain architecture**	**Bulk**		**Particle**		
	***E***_**T **_**(GPa)**	***E***_**C **_**(GPa)**	***E***_**C1 **_**(MPa)**	***E***_**C2 **_**(MPa)**	***E***_**C4 **_**(MPa)**
Linear	1.29	1.32	13.2	53.9	905.6
Branched	1.19	1.43	19.6	85.2	926.0
Network	1.80	2.01	34.6	92.0	1,270.4

**Figure 3:**
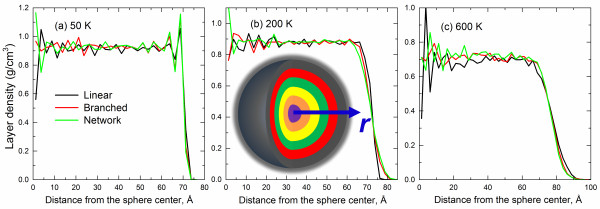
**Density profiles of PE particles at various temperatures. (a)** 50 K, **(b)** 200 K, and **(c)** 600 K, respectively.

Density profiles are effective tools to distinguish the surface and core regions of nanoparticles. To obtain the local mass density, the PE particles were partitioned into spherical shells with a thickness of 2.5 Å, extending from the center of the particle, as illustrated by the inset of Figure 3b. The number of beads that fall into each shell is counted, and the total mass in each shell is then calculated. Thus, the local density for each shell is obtained by dividing their mass by the volume. Representative density profiles for PE particles at three distinct temperatures are displayed in Figure 3. The obvious fluctuation in density close to *r* = 0 resulted from poorer statistical sampling for shell bins of small radius. As the distance from the center of the sphere increases, the particle density is identical with the bulk PE. Approaching the surface, the local density follows a sigmoidal profile, suggesting the presence of surface layering. Similar density profiles with the sigmoidal feature of the surface have been also observed in a simulated PE melt/graphite interface system [33,34]. For this discussion, the interfacial thickness is defined by the distance over which the mass density falls from its bulk value to nearly zero. The polymer chains in this region have more mobility than those in the particle. From Figure 3, it is clear that interfacial thickness increases with increasing thermal motion. Specifically, a thickness of around 5 Å is observed at 50 K, while a thickness of 25 Å is evident at 600 K. Daoulas et al. [34] reported a thickness of around 20 Å for a PE film at 400 K via both MD and MC simulations. The relatively sharp interface suggests that the PE particle has an ultrafine spherical shape. There is a tendency for beads to segregate at the surface at low temperatures, similar to the study by Mansfield and Theodorou [35] in which Monte Carlo simulations were used to predict strong temperature-dependent structural properties. From Figure 3, it is evident that the interfacial thickness is independent of the chain architecture.

**Figure 4:**
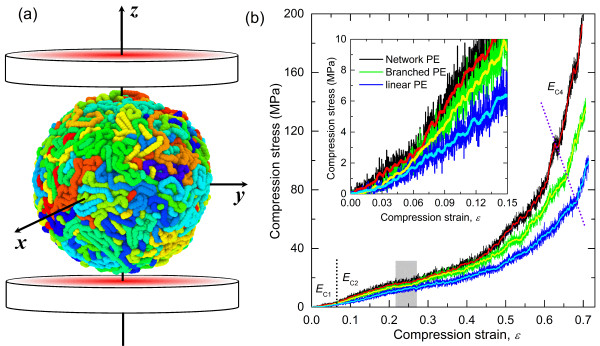
**Compression stress and compression strain. (a)** Schematic of the compression simulation of nanoscale PE particles. Beads are colored according to the molecular number. **(b)** Compressive stress–strain behaviors of PE nanoparticles with different molecular structures. Bold lines are the average of particle response.

For the flat-punch MD simulations, rigid plates were placed at the top and bottom of the prepared PE particle model with a gap of 5 Å, as depicted in Figure 4a. To eliminate the influence of initial adhesion due to molecular interaction of spherical particles with the rigid plate, only repulsive forces were assigned between the plates and the particle beads. The repulsive forces between the plates and the beads were also defined by Equation 2 with the same specified force constant *K*. However, *R* is the position of plates, and *r − R* is the distance from plates. When the beads fall outside of the region between the two plates, the repulsive forces equal to zero. Both plates were displaced toward the particle center with a constant velocity of 1 m/s (identical to compression strain rate of the bulk case) to compress the particle. Compression simulations were performed at 200 K under the NVT ensemble controlled by a Nosé-Hoover thermostat [30]. With the absence of attractive interactions between the particle and punch plates, the particles exhibited rigid rotations during the simulations. Once the compression strain increased to a critical level, the confinement by the plates restricted the particle rotation. The detailed compression process for the branched PE particle is shown in video 1 of Additional file 1. The nominal compression stress and strain are respectively determined by:

(4) $$\sigma_{c}=\frac{P_{\text{plate}}}{\pi R_{\text{particle}}^{2}},$$

(5) $$\epsilon_{c}=\frac{D-D_{0}}{R_{\text{particle}}},$$

where *R*_particle_ is the initial radius of particle, *P*_plate_ is the total reactive force of beads onto the plate, *D* is the displacement of the plate, and *D*_0_ is the gap distance between the plate and particle prior to compression. Figure 4b presents the nominal compression stress–strain curves of the PE particles with different chain architectures. In general, highly nonlinear stress–strain behaviors are observed which resulted from the change in contact area during the simulation as well as the usual increase in hydrostatic loading during compression, similar to experimental observations [19-21]. Four different regimes of compression behaviors can be identified from Figure 4b. In the first regime, it is observed that the slope of the compression stress–strain curve has a sudden change at a strain around 0.06. This regime is primarily associated with the compression of the outer surface of the particle, which has a mass density that is lower than the inner bulk-level density and a depth of the interfacial thickness. As the applied deformation approaches a strain of 0.06, this lower density region becomes highly compressed and the overall compressive load starts transferring to the denser material under the surface. The second regime begins with the sudden increase in load due to this transfer of load to the denser subsurface. This behavior in this regime is similar to that observed in the initial phase of compression of micron-sized polymeric particles [19-21], in which the ratio of surface thickness to radius is very small. The third regime is associated with brief window strain softening, as indicated by the gray-shaded region in Figure 4b. This behavior is caused by an increase in molecular rearrangements that serve to temporarily relax the applied compressive load. In the fourth regime, significant hardening occurs that is typical of uniaxial compression testing of polymers. This hardening is associated with the buildup of hydrostatic compressive forces within the particle. The effective compression moduli from the first, second, and fourth regimes were obtained by fitting the initial linear portions of the curves and are listed in Table 2. Comparison of these moduli for different chain architectures for each regime indicates that the stiffness of the network polymeric particle is consistently higher than that of the branched particle, which is consistently higher than that of the linear chain particle for all of the regimes. Therefore, the chain architecture plays a leading role on the compression behavior of PE nanoparticles.

**Figure 5:**
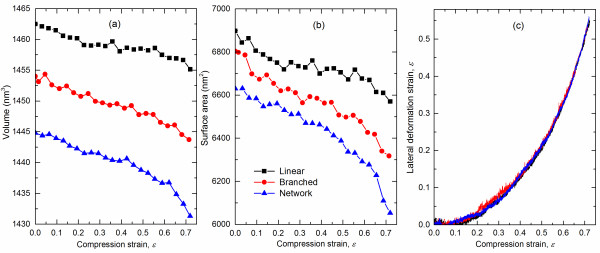
**Volume (a), surface area (b), and lateral strain (c) of PE nanoparticles.** As a function of compression strain.

The overall shape and size of the polymeric nanoparticles during the compression process are indicators of the molecular deformation mechanisms occurring within. The volume and surface area of the nanoparticles were calculated during the compression process using a tool available with the Materials Studio (Accelrys, Inc., San Diego, CA, USA) modeling package. Figure 5a,b shows the volume and surface area of the nanoparticles as a function of applied compression strain, respectively. Overall, both volume and surface area decrease with increasing levels of strain for the three chain architectures. This indicates that densification occurs during the whole compression process, independent of the chain architecture. However, the chain architecture influences the initial and deformed volumes and surface areas of the deformed nanoparticles. In the undeformed state, the networked molecules have a more compact structure compared to the other two and demonstrate a larger compressibility during deformation. This behavior originates from the relatively low mobility of the cross-linked network chains. Several local changes of volume and surface area in the curves indicate a complex deformation process that includes stepwise chain slipping and large configurational changes to relax the strain energy. At very large deformations, a steep decrease of volume and surface area appears, which corresponds to the fourth regime of the compressive stress–strain curves in Figure 4b. The lateral extension strain of the compressed nanoparticles versus the applied compressive strain for each of the three chain architectures is shown in Figure 5c. The negligible lateral extension strain below an applied compressive strain of 0.06 corresponds to the first deformation regime, thus confirming the compression of the low-density surface region. From Figure 5c, it is clear that the chain architecture plays an insignificant role on the lateral deformation of the nanoparticles for the entire range of applied compressive strains.

**Figure 6:**
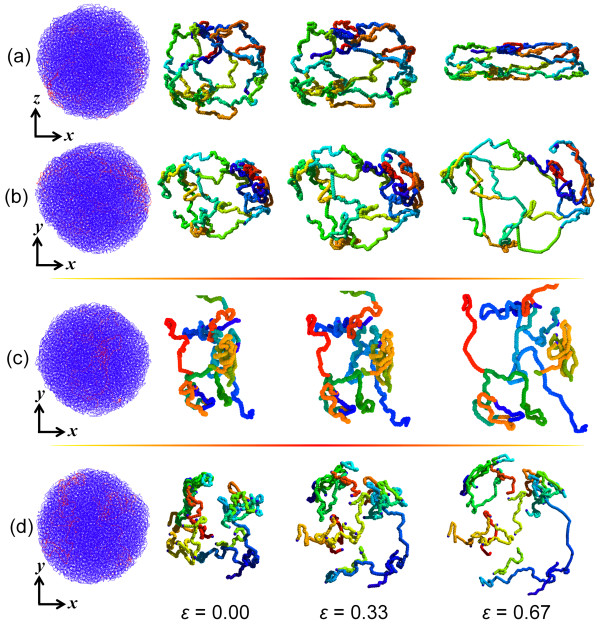
**Representative molecular snapshots at different compression strain levels. (a, b)** Side and top views of typical networked molecules in polymeric particle, respectively. **(c, d)** Top view of branched and linear chains in polymeric particles, respectively. The red-highlighted chains in the particles (left side of figure) correspond to those shown for each strain level.

Visualization of the PE chains in the nanoparticles during the compression loading process helps to reveal the molecular deformation mechanisms. Figure 6 shows representative three-dimensional (3D) molecular configurations extracted from the simulation of nanoparticle systems at different compressive strains. The selected molecules exhibit kinking and physical entanglement. Figure 6a, b presents side and top views, respectively, of distinct changes in the network chain conformation during the compression process. Specifically, as shown in Figure 6a, the network chain undergoes significant realignment due to the contraction in *z* direction and expansion in *x* direction. However, from Figure 6b, the network expands in the *x*-*y* plane when compressed in the *z* direction. The area of the network loops increases with increasing compression strain, and the molecular segments in the loops approach full extension. From Figure 6c, the branched molecular segments are disengaged throughout the compression process. This happens to a larger extent to the linear chains, as shown in Figure 6d.

## Conclusions

MD models of ultrafine monodisperse polymeric nanoparticles with networked, branched, and linear chain architectures were developed using simulated spherical hydrostatic compression of groups of coarse-grained PE molecules. The mechanical response of these nanoparticles subjected to a simulated flat-punch compression test was predicted and compared to that predicted from a 3D bulk simulation of PE. It was determined that the network configuration yielded stronger nanoparticles than those with branched or linear chain configurations. These findings were consistent with the predictions of the bulk PE models. It was also shown that the nanoparticles have a relatively uniform mass density and that individual chains have unique morphologies for high values of compression for the three different architecture types. The results of this study are important for the understanding of chain architecture on the behavior of polymeric nanoparticles that are used in a wide range of engineering applications. The mechanical properties of these particles can be tailored to specific levels simply by adjusting the chain architecture between network, branched, and linear systems. While it is evident that the network architecture yields nanoparticles with a stiffer response, the linear system results in nanoparticles with lower compressive loads for a given compressive strain.

## Competing interests

The authors declare that they have no competing interests.

## Authors’ contributions

ZZ conceived the research framework. JW carried out all the atomistic simulations and drafted the manuscript. JH, GO, and ZZ participated the analysis of the data and proofread the manuscript. All authors read and approved the final manuscript.

## Supplementary Material

Additional file 1 Supplementary material contains one video that records the compression process of a branched PE nanoparticle. Click here for file
